# Nutrition, lifestyle and colorectal cancer incidence: a prospective investigation of 10 998 vegetarians and non-vegetarians in the United Kingdom

**DOI:** 10.1038/sj.bjc.6601441

**Published:** 2004-01-06

**Authors:** M A Sanjoaquin, P N Appleby, M Thorogood, J I Mann, T J Key

**Affiliations:** 1 Cancer Research UK, Epidemiology Unit, University of Oxford, Oxford OX2 6HE, UK; 2 Warwick Medical School, University of Warwick, Coventry, CV4 7AL, UK; 3 Department of Human Nutrition, University of Otago, Dunedin, New Zealand

**Keywords:** colorectal cancer, vegetarian, nutrition, incidence, smoking

## Abstract

In a cohort of 10 998 men and women, 95 incident cases of colorectal cancer were recorded after 17 years. Risk increased in association with smoking, alcohol, and white bread consumption, and decreased with frequent consumption of fruit. The relative risk in vegetarians compared with nonvegetarians was 0.85 (95% CI: 0.55–1.32).

In Europe and most of the industrialised world, colorectal cancer is the third most common cancer in men after lung and prostate cancer and the second most common in women after breast cancer (Parkin, 2001). A genetic component of risk is well established (Cannon-Albright *et al*, 1988), but diet is widely thought to be the most important determinant of risk. Two major reports reviewed the association between meat consumption and colorectal cancer risk and agreed that the findings were inconsistent, but suggestive of a positive association (World Cancer Research Fund, 1997; COMA, 1998). Evidence that risk is reduced by a relatively high intake of fruit and vegetables, and/or dietary fibre, is suggestive but not conclusive (Fuchs *et al*., 1999; Michels *et al*., 2000; Terry *et al*, 2001; Bingham *et al*, 2003).

We aimed to examine the relationship of lifestyle and dietary factors with the incidence of colorectal cancer in a cohort that included a large proportion of vegetarians. In particular, we sought to examine whether the risk for colorectal cancer is lower in vegetarians than in meat-eaters, and low in participants who reported consuming relatively large amounts of fruit or vegetables and other foods high in fibre.

## SUBJECTS AND METHODS

The Oxford Vegetarian Study (Appleby *et al*, 1999) is a prospective investigation of 11 140 vegetarians and nonvegetarians who were recruited in the United Kingdom between 1980 and 1984. Participants were contacted through the Vegetarian Society of the United Kingdom, publicity in the national and local media, and word of mouth via participants already recruited. Non-vegetarian participants were recruited by the vegetarian participants, who were asked to nominate friends and relatives who ate meat, fish, or both.

Upon entry in to the study, participants completed a questionnaire including a simple food frequency questionnaire. Questions on other lifestyle factors related to health (smoking, alcohol consumption, and amount of exercise), date of birth, occupation, height and weight, and medical history (including illnesses related to the risk of cardiovascular disease and, for women, reproductive history) were also included. The validity of the questionnaire has been examined for estimating dietary fibre intake, but not for other nutrients (Gear *et al*, 1979). Participants were categorised into tertiles of the distribution of intake of total fat from animal foods (meat, eggs, milk, and cheese), as well as for dietary fibre derived from cereals, fruit, and vegetables. Participants were classified as vegetarians (including lacto-ovo-vegetarians and vegans) or nonvegetarians (meat eaters and people who ate fish but not meat), using their answers to questions on the consumption of meat, fish, dairy products, and eggs.

Each participant was flagged at the UK National Health Service central register and participants were followed for information on cancer registration and death. Participants were included in this analysis if they were aged 16–89 years at entry, had not been diagnosed with a malignant cancer before recruitment (except for nonmelanoma skin cancer, ICD9 code 173), and could be classified according to their smoking status and alcohol consumption. Participants were followed up to 31 December 1999, subject to censoring at age 90.

Cox's proportional hazards model was used to estimate the association between selected nutritional and lifestyle factors and the risk of colorectal cancer. All incidence rate ratios were adjusted for age at recruitment (in 11 categories: <40, 40–44, 85–89 years) and sex. Further adjustments were made for smoking status (in three categories: never, former, and current smoker) and alcohol consumption (in three categories: non-/occasional drinker, 1–7 u week^−1^ and >7 u week^−1^). The statistical analysis was performed using the STATA statistical package (StatCorp. 2001).

## RESULTS

A total of 10 998 participants were included in the analysis with an average follow-up of 17 years. There were 95 incident colorectal cancer cases, 39 in vegetarians and 56 in nonvegetarians. Table 1

**Table 1:** Baseline characteristics of the participants by sex, given as number (percentage) of participants except where indicated

**Characteristic**	**Men (*n*=4162)**	**Women (*n*=6836)**	**Total (*n*=10998)**
Median age at entry (years)	34	33	33

*Smoking*			
Never smoker	1816 (43.6)	4103 (60.0)	5919 (53.8)
Former smoker	1317 (31.7)	1618 (23.7)	2935 (26.7)
Current smoker	1029 (24.7)	1115 (16.3)	2144 (19.5)

*Alcohol*			
<1 unit/week	948 (22.8)	2223 (32.5)	3171 (28.8)
1–7 units/week	1194 (28.7)	3101 (45.4)	4295 (39.1)
>7 units/week	2020 (48.5)	1512 (22.1)	3532 (32.1)

*Body mass index*^a^*(kg m*^−*2*^*)*			
<20	594 (14.4)	1781 (26.6)	2375 (22.0)
20–<22.5	1567 (38.1)	2806 (41.9)	4373 (40.4)
22.5–<25	1261 (30.6)	1407 (21.0)	2668 (25.0)
25+	693 (16.8)	706 (10.5)	1399 (13.0)
Median	22.4	21.4	21.7

*Diet group*			
Non–vegetarians	2565 (61.6)	3780 (55.3)	6345 (57.7)
Vegetarians	1597 (38.4)	3056 (44.7)	4653 (42.3)

*Median dietary fibre intake*^b^*(g day*^−*1*^*)*			
Bottom third	17.9	16.5	17.0
Middle third	27.3	24.7	25.7
Top third	39.6	35.0	36.7

*Median animal fat intake*^c^*(g day*^−*1*^*)*			
Bottom third	25.5	23.6	24.8
Middle third	52.4	45.2	47.2
Top third	74.7	67.0	70.6


a Body mass index is unknown for 183 participants.

b Dietary fibre intake (Southgate fibre) is unknown for 3052 participants.

c Animal fat intake is unknown for 1470 participants.

shows the baseline characteristics of the participants. Median age at entry was 34 years for men and 33 for women. In all, 38% of men and 45% of women were vegetarians. The Standardized Incidence Ratio (SIR) for colorectal cancer compared to the general population of England and Wales was 0.91 (95% CI: 0.74–1.12). The SIRs for vegetarians and non-vegetarians were 0.81 (95% CI: 0.58–1.11) and 1.00 (95% CI: 0.76–1.30), respectively.

Table 2

**Table 2:** Relative risks (95% CI) for colorectal cancer associated with selected dietary and lifestyle factors

**Factor**	**Category**	**Cases^a^**	**Relative risk^b^**	***P*^c^**	**Relative risk^d^**	***P*^c^**
Sex	Male	37	1.00		1.00	
	Female	58	0.85 (0.56–1.29)	0.452	1.02 (0.66–1.56)	0.941

Diet group	Non-vegetarians	56	1.00		1.00	
	Vegetarians	39	0.72 (0.48–1.10)	0.132	0.85 (0.55–1.32)	0.463

Meat	Not eaten	48	1.00		1.00	
	Eaten less than daily	21	1.35 (0.80–2.27)		1.19 (0.70–2.02)	
	Eaten daily	24	1.34 (0.81–2.23)	0.209	1.14 (0.67–1.93)	0.581

Fish	Not eaten	40	1.00		1.00	
	Less than once/week	23	1.41 (0.84–2.37)		1.21 (0.71–2.06)	
	Once or more/week	32	1.38 (0.86–2.22)	0.168	1.17 (0.71–1.92)	0.530

Eggs	<1 eggs week^−1^	15	1.00		1.00	
	1–5 eggs week^−1^	58	1.32 (0.75–2.33)		1.24 (0.70–2.20)	
	6+ eggs week^−1^	21	1.43 (0.73–2.78)	0.301	1.29 (0.66–2.52)	0.428

Milk	<0.5 pints day^−1^	31	1.00		1.00	
	0.5 pints day^−1^	36	0.88 (0.54–1.42)		0.86 (0.53–1.40)	
	>0.5 pints day^−1^	26	1.08 (0.64–1.84)	0.809	1.10 (0.65–1.87)	0.779

Cheese	<5 times week^−1^	41	1.00		1.00	
	5 to 9 times week^−1^	42	1.26 (0.82–1.94)		1.26 (0.82–1.94)	
	10+ times week^−1^	9	1.02 (0.49–2.10)	0.580	0.98 (0.48–2.03)	0.631

Fresh or dried fruit	<5 times week^−1^	31	1.00		1.00	
	5 to 9 times week^−1^	33	0.58 (0.35–0.95)		0.59 (0.36–0.98)	
	10+ times week^−1^	27	0.57 (0.34–0.97)	0.041	0.60 (0.35–1.02)	0.067

Total vegetables	Lowest third	46	1.00		1.00	
	Middle third	19	0.52 (0.30–0.89)		0.53 (0.31–0.90)	
	Highest third	30	0.85 (0.53–1.34)	0.357	0.86 (0.54–1.38)	0.415

Brown bread	<15 slices week^−1^	45	1.00		1.00	
	15+ slices week^−1^	44	0.86 (0.56–1.31)	0.482	0.90 (0.59–1.38)	0.638

White bread	<15 slices week^−1^	43	1.00		1.00	
	15+ slices week^−1^	16	2.25 (1.25–4.04)	0.006	2.11 (1.17–3.81)	0.009

Breakfast cereals	Not eaten	25	1.00		1.00	
	<5 times week^−1^	15	1.08 (0.57–2.05)		1.12 (0.59–2.13)	
	5+ times week^−1^	47	1.14 (0.70–1.85)	0.606	1.24 (0.76–2.03)	0.389

Total dietary fibre	Lowest third	20	1.00		1.00	
	Middle third	24	1.03 (0.57–1.87)		1.07 (0.59–1.95)	
	Highest third	19	0.73 (0.39–1.37)	0.300	0.82 (0.43–1.56)	0.424

Total animal fat	Lowest third	23	1.00		1.00	
	Middle third	33	1.65 (0.97–2.81)		1.55 (0.91–2.66)	
	Highest third	20	1.16 (0.64–2.13)	0.528	1.07 (0.58–1.97)	0.660

Vitamin supplements	Not used	65	1.00		1.00	
	Used	26	1.02 (0.65–1.62)	0.925	1.00 (0.63–1.59)	0.993

Smoking	Never smoker	36	1.00		1.00	
	Former smoker	43	1.95 (1.24–3.07)		1.80 (1.13–2.85)	
	Current smoker	16	1.88 (1.03–3.44)	0.009	1.70 (0.92–3.15)	0.034

Alcohol	<1 unit week^−1^	30	1.00		1.00	
	1–7 units week^−1^	39	1.69 (1.04–2.74)		1.53 (0.94–2.49)	
	>7 units week^−1^	26	1.81 (1.04–3.15)	0.025	1.53 (0.87–2.69)	0.118

Social class	I–II	29	1.00		1.00	
	III–V	24	1.46 (0.85–2.52)	0.183	1.44 (0.83–2.48)	0.161
	Other/unknown	42	0.97 (0.53–1.76)		0.98 (0.54–1.78)	

Exercise^e^	Low	76	1.00		1.00	
	High	19	0.82 (0.49–1.37)	0.453	0.82 (0.49–1.36)	0.440

Body mass index (kg m^−2^)	<20	17	1.00		1.00	
	20−<22.5	23	0.72 (0.38–1.34)		0.69 (0.37–1.29)	
	22.5−<25	38	1.48 (0.83–2.66)		1.37 (0.76–2.46)	
	25+	14	0.83 (0.40–1.70)	0.535	0.74 (0.36–1.53)	0.791

a The total number of cases does not always equal 95 because the level of the factor may be unknown for some cases.

b Adjusted for age and sex.

c *P* for trend (or heterogeneity between categories for sex and smoking). The test of linear trend simply ranks the categories 1, 2, 3 etc. and excludes the other or unknown category for social class.

d Adjusted for age, sex, alcohol and smoking.

e High exercise is defined as sport, keep fit, running or cycling at least twice a week.

shows relative risks (RRs) and confidence intervals, nutritional and lifestyle factors, and colorectal cancer risk, adjusted for age and sex alone and with further adjustment for smoking and alcohol. Vegetarians showed a moderately but nonsignificantly lower risk of colorectal cancer compared with the nonvegetarians (RR 0.72, 95% CI: 0.48–1.10), but this association became weaker after adjusting for smoking and alcohol (RR 0.85, 95% CI: 0.55–1.32). Among the nonvegetarians, there was no evidence of a positive association with the frequency of meat consumption. Among the other dietary factors, the only statistically significant associations with risk were for fruit and white bread consumption. Participants with the highest consumption of fresh or dried fruit experienced a reduction of colorectal cancer risk (RR 0.57, 95% CI: 0.34–0.97, *P* for trend=0.041), although the association was no longer statistically significant after adjusting for smoking and alcohol. Participants eating 15 or more slices of white bread per week compared with those eating less than 15 had significantly higher risk (RR=2.25, 95% CI: 1.25–4.04; *P* for difference between groups=0.006), which remained highly significant after adjusting for alcohol and smoking.

After adjusting for alcohol intake, both current and former smokers had an increased risk of colorectal cancer compared with the never smokers (RR=1.70, 95% CI: 0.92–3.15 and RR=1.80, 95% CI: 1.13–2.85, respectively). Among the other lifestyle factors, social class, exercise, alcohol consumption, and body mass index were not significantly associated with the risk of colorectal cancer.

## DISCUSSION

This prospective study had a wide variation in diet due to the inclusion of a large proportion of vegetarians. The main limitation is the relatively small number of colorectal cancer cases and the lack of sophistication of the food frequency questionnaire.

The present analysis did not find a significant difference in risk between nonvegetarians and vegetarians. Furthermore, no increase in risk of colorectal cancer was seen with higher meat consumption among nonvegetarians. Nevertheless, the lack of statistical association may reflect the relative small number of cases. A previous analysis of mortality in this cohort (Appleby *et al*, 2002) showed similar death rates for colorectal cancer in vegetarians and non-vegetarians based on 25 and 24 deaths from colorectal cancer, respectively. However, in a prospective investigation of Seventh-day Adventists (Singh and Fraser, 1998), cancer of the colon was significantly more common in non-vegetarians than in vegetarians. It could be suggested that the nonvegetarians in our study represent a healthy group compared with the population at large, and that this might account for the lack of difference between the vegetarians and non-vegetarians; however, the SIR among non-vegetarians was exactly one.

Fresh or dried fruit consumption was found to be significantly associated with colorectal cancer risk, although this association became nonsignificant after adjusting for alcohol and smoking. An approximately 40% decrease in risk was seen in people eating fresh or dried fruit five or more times per week compared with persons eating less than this amount. We did not observe a significant association for brown bread and risk of colorectal cancer, but a two-fold increase in risk was detected in those consuming 15 or more slices of white bread per week. White bread consumption may be a marker of an unhealthy diet, although an adverse association of refined carbohydrates with risk has been noted before (Chatenoud *et al*, 1999). We did not observe a significant association between fibre and colorectal cancer risk; however, information needed to estimate dietary fibre intake was unavailable for 32 cases, and the results are compatible with a recent report of a reduction in risk with high fibre intake (Bingham *et al*, 2003).

Our study suggested that smoking was associated with an almost two-fold increase in risk of colorectal cancer, although this association was attenuated by adjusting for alcohol consumption. The apparent adverse effect of alcohol was also partially confounded by smoking. Both the WCRF report (World Cancer Research Fund, 1997) and a comprehensive review by Potter (1999) concluded that smoking and alcohol are probable risk factors for colorectal cancer.
